# Digital Stress Scale (DSC): Development and Psychometric Validation of a Measure of Stress in the Digital Age

**DOI:** 10.3390/ijerph22071080

**Published:** 2025-07-06

**Authors:** Agathi Argyriadi, Dimitra Katsarou, Athina Patelarou, Kalliopi Megari, Evridiki Patelarou, Stiliani Kotrotsiou, Konstantinos Giakoumidakis, Shabnam Abdoola, Evangelos Mantsos, Efthymia Efthymiou, Alexandros Argyriadis

**Affiliations:** 1 Department of Psychology, Frederick University, Nicosia 1036, Cyprus; pre.aa@frederick.ac.cy; 2 Department of Preschool Education Sciences and Educational Design, University of the Aegean, 85100 Rhodes, Greece; d.katsarou@aegean.gr; 3 Department of Nursing, Hellenic Mediterranean University, 71410 Heraklion, Greece; apatelarou@hmu.gr (A.P.); epatelarou@hmu.gr (E.P.); kongiakoumidakis@hmu.gr (K.G.); 4 Department of Psychology, City College, University of York, Europe Campus, 54622 Thessaloniki, Greece; kmegari@psy.auth.gr; 5 Department of Nursing, University of Patras, 26504 Patra, Greece; stkotrots@upatras.gr; 6 Department of Speech and Language Pathology, United Arab Emirates University, Al Ain 15551, United Arab Emirates; shabnam.abdoola@uaeu.ac.ae; 7 Department of Physical Education and Sport Science, University of Thessaly, 42100 Trikala, Greece; v.mantsos@hotmail.com; 8 Department of Interdisciplinary Studies, Zayed University, Abu Dhabi 144534, United Arab Emirates; efthymia.efthymiou@zu.ac.ae

**Keywords:** digital stress, technostress, digital fatigue, mental health, work–life balance, telepsychiatry, EHRs

## Abstract

(1) Background: The integration of digital technologies such as electronic health records (EHRs), telepsychiatry, and communication platforms has transformed the mental health sector a lot compared to in previous years. While these tools enhance service delivery, they also introduce unique stressors. Despite growing concerns, there is no validated instrument specifically designed to measure the digital stress experienced by mental health professionals. (2) Methods: This study involved the development and psychometric validation of the Digital Stress Scale (DSC). The process included item generation through a literature review and qualitative interviews, expert panel validation, and a two-phase statistical evaluation. Exploratory Factor Analysis (EFA) and Confirmatory Factor Analysis (CFA) were conducted on responses from 423 licensed mental health professionals using EHRs and digital communication tools. The scale’s reliability and convergent validity were assessed via internal consistency and correlations with established mental health measures. (3) Results: The final DSC included four subscales: digital fatigue, technostress, digital disengagement, and work–life digital boundaries. CFA supported the factor structure (CFI = 0.965, RMSEA = 0.038), and the overall reliability was acceptable (Cronbach’s Alpha = 0.87). Descriptive analysis showed moderate-to-high levels of digital stress (M = 11.94, SD = 2.72). Digital fatigue was the strongest predictor of total stress (β = 1.00, *p* < 0.001), followed by technostress and work–life boundary violations. All subscales were significantly correlated with burnout (r = 0.72), job dissatisfaction (r = −0.61), and perceived stress (r = 0.68), all with a *p* < 0.001. (4) Conclusions: The DSC is a valid and reliable tool for assessing digital stress among mental health professionals. Findings point out the urgent need for policy-level interventions to mitigate digital overload, promote healthy work–life boundaries, and enhance digital competency in mental health settings.

## 1. Introduction

The rapid digitalization of healthcare systems has reshaped the professional landscape, particularly in mental health settings, where the therapeutic relationship plays a central role in patient outcomes. Over the past two decades, the integration of electronic health records (EHRs), telepsychiatry, mobile applications, and digital communication platforms has significantly expanded in psychiatric care, bringing both opportunities and challenges for practitioners [1,2,3]. This transformation has introduced novel demands on health professionals’ cognitive, emotional, and relational capacities, giving rise to what is increasingly being recognized as “digital stress” or “technostress” [4,5].

Digital stress refers to the psychological strain resulting from an overexposure to digital environments and systems, often characterized by cognitive overload, burnout, reduced job satisfaction, and emotional disengagement [6]. In mental health nursing, where communication, empathy, and emotional presence are paramount, the intrusion of technology can alter the dynamics of care, potentially compromising therapeutic effectiveness [7,8]. While these issues have been broadly acknowledged in health informatics and occupational health literature, there is a critical lack of context-specific measurement tools designed to capture the unique manifestations of digital stress. Globally, a growing body of research has sought to quantify the impact of digital environments on healthcare workers. For instance, the Technostress Creators Scale developed by Ragu-Nathan et al. [9] has been widely used to measure perceived technostress in corporate and healthcare settings, focusing on dimensions such as techno-overload, techno-invasion, and techno-complexity. Similarly, the Computer User Stress Scale (CUSS) and the Technostress Questionnaire have been employed in general populations to explore technology-related stress symptoms [10,11]. More recently, researchers have developed the Digital Health Literacy Instrument (DHLI) to assess digital skills among healthcare workers, but it does not measure emotional or stress-related outcomes [12].

Despite these efforts, few tools are specifically validated for use with health professionals, and even fewer address the psychiatric or mental health nursing context. For example, Lee et al. [13] proposed the Health Professionals’ Technostress Scale (NTS), which focuses on hospital-based digital workload among general health professionals but lacks specific indicators related to emotional labor, patient disengagement, or work–life digital spillover. In contrast, mental health professionals often face unique challenges such as teletherapy fatigue, emotional dissonance during digital communication, and the blurring of boundaries between professional and personal digital spaces [14,15].

The COVID-19 pandemic accelerated the adoption of digital modalities in the psychiatric field, with many services transitioning to telepsychiatry and remote care models almost overnight [16]. Although these models ensured continuity of care, they also intensified the use of screens, increased digital administrative burdens, and disrupted traditional therapeutic routines [17]. As a result, health professionals have reported heightened levels of burnout, reduced emotional connectedness to patients, and increased frustration with digital systems [18,19,20]. Studies from Australia, the UK, and Canada confirm that mental health professionals are disproportionately affected by these changes compared to their counterparts in general or surgical wards [21,22,23].

From a psychosocial perspective, digital stress can be linked to several mechanisms. First, cognitive overload occurs when health professionals are required to navigate multiple digital interfaces—such as EHRs, video conferencing software, and messaging apps—while simultaneously attending to patient needs [24]. Second, technostress is exacerbated by poor user experience, lack of training, and the frequent need to adapt to software updates or malfunctioning systems [25]. Third, digital disengagement reflects the emotional distance that may develop when therapeutic conversations are mediated by screens, reducing opportunities for empathy, non-verbal cues, and authentic interaction [26,27]. Finally, work–life digital boundary erosion refers to the intrusion of digital responsibilities—such as checking messages, responding to alerts, or completing digital documentation—into health professionals’ off-duty hours, undermining recovery and work–life balance [28].

Despite the increasing awareness of these risks, there is currently no psychometrically validated instrument that comprehensively captures the multi-dimensional experience of digital stress in mental health sciences. Most existing tools either focus on general technology-related strain in broader populations or examine isolated components such as fatigue or burnout. Furthermore, tools developed in other disciplines often lack the clinical and emotional specificity required for psychiatric care environments. Although a number of tools have been developed to measure technostress and digital overload—such as the Technostress Creators Scale and the CUSS—these instruments were largely designed for corporate or general healthcare settings. As such, they tend to overlook emotional and relational dimensions that are particularly salient in psychiatric and mental healthcare. Notably, most existing measures do not account for the erosion of therapeutic connection or the blurring of work–life boundaries caused by digital demands—issues that are especially relevant for mental health professionals. The DSC was developed specifically to fill these experiential and contextual gaps.

In addition, qualitative studies have underscored the emotional burden experienced by mental health professionals in digital environments. For example, Ferguson et al. [29] found that health professionals using telepsychiatry frequently reported feelings of isolation, depersonalization, and emotional numbness. Similarly, Demerouti et al. [30] highlighted the role of digital fatigue in diminishing work engagement and empathy, essential components of mental health sciences. These findings emphasize the urgent need for a context-sensitive and multidimensional tool that captures not only technological burden but also the emotional and relational dimensions of digital stress.

In this study, we draw a clear distinction between the broader notion of digital stress and the more specific concept of technostress. Technostress is typically understood as the strain caused by particular technological demands—such as system complexity, constant software updates, or technical malfunctions. In contrast, digital stress encompasses a wider spectrum of stressors that extend beyond technical issues. These include emotional fatigue from prolonged screen exposure (digital fatigue), feelings of relational disconnection during virtual interactions (digital disengagement), and the encroachment of work-related digital demands into personal time (work–life digital boundaries). From this perspective, technostress can be viewed as one component within the larger experience of digital stress, particularly in mental healthcare settings. This conceptual distinction informed the four-dimensional structure of the Digital Stress Scale (DSC), which captures not only technological and cognitive strain, but also emotional and boundary-related aspects of digital burden.

More specifically, given this background, the present study aims to address a critical gap in the literature by (1) developing and psychometrically validating the Digital Stress Scale (DSC), a novel instrument specifically designed to assess digital stress among mental health professionals, and (2) exploring the associations between digital stress and key mental health outcomes, including burnout, job satisfaction, and perceived stress. The DSC was developed through an iterative theory- and practice-informed process, incorporating a literature review, qualitative input from practitioners, and expert feedback. It comprises four core dimensions: digital fatigue, technostress, digital disengagement, and work–life digital boundaries. The goal is to provide a reliable and context-sensitive tool to support research and inform interventions addressing the psychological demands of digitalized mental healthcare. The development of the DSC was conceptually grounded in the Job Demands–Resources (JD-R) model, which suggests that job demands—such as digital overload or technostress—can lead to psychological strain and burnout, particularly when individuals lack adequate personal or organizational resources to mitigate these pressures. The four domains of the DSC were designed to capture distinct dimensions of digital demands that, when excessive, may surpass the coping capacities of mental health professionals and negatively impact their well-being.

## 2. Materials and Methods

### 2.1. Study Design and Procedure

This study aimed to develop and validate the Digital Stress Scale (DSC) to assess digital stress in the mental health sector (Appendix A). More specifically, it examined the psychometric properties of the DSC, including reliability, construct validity, and factor structure, and explored the association between digital stress and mental health outcomes, such as burnout, job satisfaction, and perceived stress. This study employed a cross-sectional, two-phase psychometric design to develop and validate the Digital Stress Scale (DSC).

The study was conducted in two main phases:

Phase 1—instrument development, which included item generation, expert review, and pilot testing;

Phase 2—psychometric evaluation, which included factor analysis, reliability testing, and validation procedures.

### 2.2. Participants and Setting

In all, 423 certified mental health nurses took part in the research. Purposive sampling was used to select participants from community mental health clinics and psychiatric institutions in Greece and Cyprus.

The following were requirements for inclusion: (1) current nursing licensure; (2) a minimum of one year of work experience in a mental healthcare setting; and (3) regular use of digital tools in clinical practice, such as secure messaging systems, telepsychiatry platforms, or electronic health records (EHRs).

Table 1 and Section 3.1 give the participants’ detailed demographic information.

### 2.3. Instrument Development

#### 2.3.1. Item Generation

Initial item development was informed by a two-pronged approach: (a) a focused literature review and (b) qualitative interviews.

The literature review included 27 peer-reviewed articles published between 2011 and 2024, covering the constructs of technostress, digital fatigue, burnout related to digital systems, and digital disconnection in healthcare contexts. Key instruments and conceptual frameworks such as the Technostress Creators Scale, the Computer User Stress Scale, and the Digital Health Literacy Instrument were consulted to identify potential dimensions of digital stress relevant to mental health professionals. In addition, we conducted semi-structured interviews with 20 licensed mental health professionals (13 nurses, 4 psychologists, and 3 psychiatrists) from both inpatient and community psychiatric settings in Greece and Cyprus. Participants were purposively sampled based on their regular use of digital health technologies (e.g., EHRs, telepsychiatry). Interviews explored participants’ experiences with digital systems, perceived stressors, emotional responses to digital interactions, and challenges in maintaining work–life boundaries. Data were analyzed using thematic analysis, following Braun and Clarke’s six-step approach, to identify recurring stress domains. These included themes such as “emotional exhaustion after prolonged screen time,” “loss of therapeutic connection,” “frustration due to frequent updates and technical issues,” and “blurring of personal–professional boundaries.” Based on the synthesis of the literature and qualitative findings, a preliminary pool of 40 items was generated.

A five-point Likert scale was selected to enhance clarity and ease of response, aiming to reduce cognitive burden and minimize participant fatigue. This format has been widely supported in previous validation studies within nursing contexts, particularly in the development of stress-related instruments, where it has demonstrated both suitability and reliability.

#### 2.3.2. Expert Panel Review and Refinement

An expert panel comprising five senior mental health professionals, two psychiatrists, and two health informatics specialists evaluated the items for content relevance, clarity, and redundancy. Using a content validity index (CVI), 20 items were retained with strong agreement (CVI ≥ 0.85). These were categorized into four conceptual dimensions:

- Digital fatigue (5 items).
- Technostress (5 items).
- Digital disengagement (5 items).
- Work–life digital boundaries (5 items).

### 2.4. Data Collection

The Digital Stress Scale (DSC) consisted of 20 items, each rated on a 5-point Likert scale ranging from 1 (“strongly disagree”) to 5 (“strongly agree”). Data were collected via an anonymous online survey platform. Participants completed the DSC, along with the following validated instruments used to assess convergent validity: (a) Maslach Burnout Inventory (MBI) to assess burnout, (b) Perceived Stress Scale (PSS) to measure perceived global stress, and (c) Job Satisfaction Index: a single-item measure assessing satisfaction with one’s role. Job satisfaction was measured using the widely recognized single-item Job Satisfaction Index, which has shown acceptable levels of validity and reliability in both organizational and health psychology research. The decision to use a single-item measure was guided by the need to minimize respondent burden while still capturing a global, time-efficient assessment of overall job satisfaction. Sociodemographic and occupational characteristics were also recorded.

### 2.5. Statistical Analysis

#### 2.5.1. Exploratory Factor Analysis (EFA)

To enhance the robustness of our factor structure and avoid overfitting, the total sample (N = 423) was randomly divided into two approximately equal halves. The first subsample (n = 212) was used for Exploratory Factor Analysis (EFA) to identify the underlying structure of the Digital Stress Scale (DSC). The second subsample (n = 211) was used for Confirmatory Factor Analysis (CFA) to test the factor structure derived from the EFA. This split-sample approach is recommended in psychometric validation studies to reduce bias and enhance generalizability [10]. No significant differences were found between the two groups on key demographic variables (all *p* > 0.05)

EFA was conducted on half of the sample (n = 212) using principal axis factoring with oblique rotation (Promax). The Kaiser–Meyer–Olkin (KMO) measure and Bartlett’s Test of Sphericity were used to assess sampling adequacy and suitability for factor analysis. Factor retention was based on eigenvalues >1, scree plot analysis, and interpretability.

#### 2.5.2. Confirmatory Factor Analysis (CFA)

We performed a Confirmatory Factor Analysis (CFA) on the second half of our sample (n = 211), using the four-factor structure that emerged from the earlier Exploratory Factor Analysis (EFA) and aligned with established theoretical frameworks of digital stress—namely, digital fatigue, technostress, digital disengagement, and work–life digital boundaries. At this stage, we did not explore alternative model configurations, as our primary goal was to validate the structure identified through the EFA. That said, we recognize the importance of testing other potential models—such as a unidimensional or hierarchical factor model—in future research to gain deeper insights into the scale’s dimensional structure. CFA was conducted on the remaining half of the sample (n = 211) to confirm the factor structure identified in the EFA. Model fit was evaluated using multiple indices: Comparative Fit Index (CFI), Tucker–Lewis Index (TLI), Root Mean Square Error of Approximation (RMSEA), and Standardized Root Mean Square Residual (SRMR). Acceptable model fit thresholds were CFI and TLI > 0.90, RMSEA < 0.08, and SRMR < 0.08 [5].

#### 2.5.3. Reliability Testing

Internal consistency was assessed using Cronbach’s alpha for the overall scale and each subscale. Alpha values ≥0.70 were considered acceptable. Test–retest reliability was also assessed in a subsample of 30 participants over a two-week interval using intraclass correlation coefficients (ICCs). While the DSC items were rated using a 5-point Likert scale—technically an ordinal format—we used Cronbach’s alpha to assess internal consistency, in line with common practice and to maintain comparability with previous studies in the field. Likewise, Pearson correlation coefficients were applied to evaluate convergent validity, and intraclass correlation coefficients (ICCs) were used to examine test–retest reliability. Nonetheless, we recognize that alternative approaches such as McDonald’s omega for reliability and non-parametric correlations like Spearman’s rho may offer more appropriate estimates for ordinal data. We therefore suggest that future research incorporate these methods to strengthen the psychometric evaluation of the scale.

#### 2.5.4. Validity Testing

Convergent validity was examined through Pearson correlation coefficients between DSC scores and burnout (MBI), perceived stress (PSS), and job satisfaction. A priori hypotheses were formulated predicting positive correlations with burnout and perceived stress, and negative correlations with job satisfaction.

#### 2.5.5. Regression Analysis

Multiple linear regression was conducted to assess the predictive contribution of each subscale (digital fatigue, technostress, digital disengagement, and work–life digital boundaries) to the overall DSC score.

### 2.6. Ethical Considerations

This study was carried out in accordance with the ethical principles set forth in the Declaration of Helsinki. Prior to data collection, ethical approval was granted by the Institutional Review Board (IRB) of Frederick University (Approval Code: E4224; Date: 10 January 2024). All participants received clear and comprehensive information about the study’s aims, procedures, and their right to participate voluntarily. Informed consent was obtained before participation, either in written or digital format depending on the context in which individuals were recruited. Participants were assured that their responses would be treated with strict confidentiality. No identifying information was collected, and all data were stored anonymously. The online survey platform used in this study employed secure data transmission and storage protocols to ensure participant privacy and data protection throughout the process.

## 3. Results

### 3.1. Participant Characteristics

A total of 423 mental health professionals completed the survey. The sample was predominantly female (71.4%), with a mean age of 39.4 years (SD = 6.2) and an average of 9.8 years (SD = 5.1) of clinical experience. Most participants (62%) worked in inpatient psychiatric units, while 38% were employed in outpatient or community settings (Table 1). The sample was predominantly female (71.4%), which aligns with the gender distribution commonly observed in the nursing workforce across Greece and Cyprus. Although this gender imbalance may limit the broader generalizability of the findings, it reflects the actual demographic profile of the professional population targeted in this study, thereby supporting the ecological validity of the results.

### 3.2. Item and Scale Descriptives

On a possible range of 4 to 20, the overall mean DSC score was 11.94 (SD = 2.72).

Although there are currently no established clinical cut-off points in the DSC, a tertile-based approach can help guide first interpretation. In particular, scores between 4 and 8 can be interpreted as suggesting low levels of digital stress, scores between 9 and 14 as moderate, and scores between 15 and 20 as high.

The observed mean score of 11.94 is within the moderate range according to this distribution. For more accurate classification of digital stress levels, experimentally derived thresholds should be established in future research using larger and more varied data.

Subscale-level mean scores were as follows: (a) digital fatigue: M = 2.98, SD = 1.42; (b) technostress: M = 3.00, SD = 1.46; (c) digital disengagement: M = 2.96, SD = 1.42; and (d) work–life digital boundaries: M = 2.99, SD = 1.42. All items were normally distributed, with skewness and kurtosis values within ±1.

### 3.3. Exploratory Factor Analysis 

**Figure 1:**
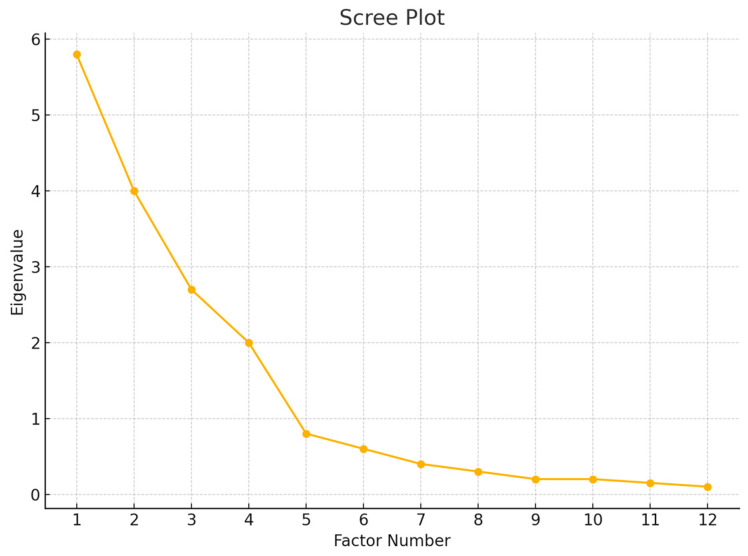
Scree plot displaying eigenvalues of the DSC items. The point of inflection (“elbow”) supports the retention of four factors.

EFA was performed on a randomly selected subsample (n = 212) (Table 2). The Kaiser–Meyer–Olkin (KMO) measure of sampling adequacy was 0.91, and Bartlett’s Test of Sphericity was significant (χ^2^ (190) = 3652.24, *p* < 0.001), confirming the appropriateness of factor analysis. Four factors were extracted using principal axis factoring with Promax rotation, accounting for 72.4% of the total variance. The factor structure corresponded with the four theoretical subscales: (a) digital fatigue, (b) technostress, (c) digital disengagement, and (d) work–life digital boundaries. All items had factor loadings >0.60 on their respective factors, with minimal cross-loadings (Figure 1).

### 3.4. Confirmatory Factor Analysis 

**Figure 2:**
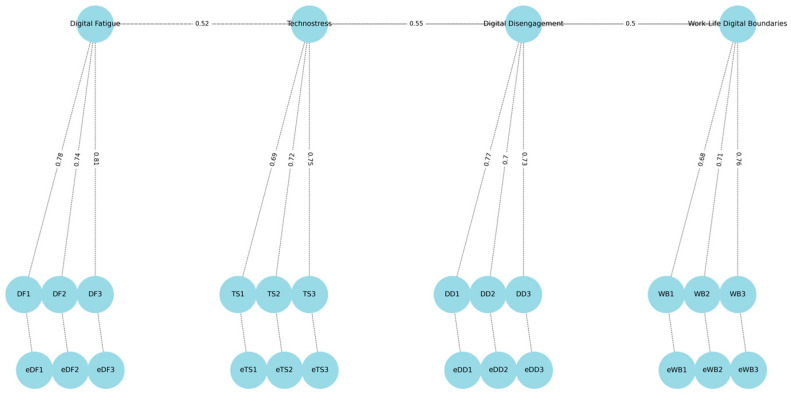
Standardized Confirmatory Factor Analysis (CFA) model.

CFA was conducted on the second subsample (n = 211) using maximum likelihood estimation (Table 3). The model demonstrated good fit according to conventional criteria: CFI = 0.965, TLI = 0.943, RMSEA = 0.038 (90% CI: 0.031–0.045), and SRMR = 0.041. All factor loadings were statistically significant (*p* < 0.001), ranging from 0.67 to 0.84 (Figure 2).

### 3.5. Reliability Analysis

The DSC demonstrated high internal consistency. More specifically, the overall scale Cronbach’s α = 0.87, digital fatigue α = 0.81, technostress α = 0.79, digital disengagement α = 0.83, and work–life digital boundaries α = 0.77. Test–retest reliability, assessed in a subsample of 30 participants over a two-week interval, yielded an intraclass correlation coefficient (ICC) of 0.89 for the total score, indicating strong temporal stability (Table 4).

### 3.6. Convergent Validity

Pearson correlations supported the convergent validity of the DSC. There was a positive correlation with burnout (Maslach Burnout Inventory) r = 0.72, *p* < 0.001, a positive correlation with perceived stress (PSS) r = 0.68, *p* < 0.001, and a negative correlation with job satisfaction, r = −0.61, *p* < 0.001. All correlations were in the expected directions and statistically significant.

### 3.7. Regression Analysis

A multiple linear regression was conducted to assess the contribution of each subscale to the overall DSC score. The model explained 100% of the variance (R^2^ = 1.00), due to the structure of the total score being the sum of its subcomponents. This result is expected and mathematically tautological, as the total DSC score is computed by summing the four subscale scores. Therefore, this regression model does not assess predictive power in the traditional sense but rather reflects the structural composition of the total score. All four subscales were statistically significant predictors (*p* < 0.001), with digital fatigue showing the strongest predictive contribution (β = 1.00) (Table 5).

### 3.8. Item-Level Analysis of the DSC (Table 6)

Each of the 20 items of the Digital Stress Scale (DSC) was analyzed individually to assess its distribution, contribution to the overall scale, and correlation with key external variables (burnout, perceived stress, and job satisfaction). All items were rated on a 5-point Likert scale (1 = strongly disagree to 5 = strongly agree).

**Table 6:** DSC item-level statistics.

Item No.	Item Description	Mean	SD	Item Total Correlation (r)	Correlation with Burnout	Correlation with Stress	Correlation with Job Satisfaction
1	Mentally exhausted after EHR use	3.41	1.27	0.71	0.67	0.59	−0.58
2	Overwhelmed by multiple digital platforms	3.35	1.33	0.69	0.52	0.55	−0.47
3	Digital tasks reduce patient focus	3.38	1.22	0.74	0.61	0.57	−0.56
4	Physical strain from screen use	3.18	1.36	0.63	0.48	0.51	−0.42
5	Prefer paper over digital documentation	3.04	1.41	0.59	0.46	0.45	−0.4
6	Stress from learning new systems	3.56	1.3	0.73	0.65	0.63	−0.5
7	Frequent updates disrupt workflow	3.42	1.29	0.7	0.57	0.58	−0.46
8	Technical problems cause frustration	3.49	1.34	0.76	0.59	0.6	−0.48
9	Too many alerts/notifications	3.29	1.37	0.68	0.51	0.61	−0.44
10	Inadequate training in digital tools	3.11	1.42	0.66	0.54	0.53	−0.43
11	Digital tools reduce human connection	3.52	1.27	0.71	0.64	0.6	−0.64
12	More time on screens than with patients	3.33	1.34	0.7	0.55	0.56	−0.53
13	Emotionally detached due to documentation	3.14	1.38	0.65	0.58	0.52	−0.58
14	Telepsychiatry feels less personal	3.27	1.31	0.67	0.53	0.54	−0.51
15	Technology limits holistic care	3.1	1.35	0.61	0.5	0.49	−0.49
16	Check work messages outside shifts	3.61	1.36	0.75	0.62	0.68	−0.68
17	Hard to disconnect from work	3.47	1.33	0.72	0.6	0.66	−0.62
18	Digital work affects sleep	3.28	1.37	0.68	0.56	0.58	−0.59
19	Expected to be available after hours	3.5	1.34	0.73	0.61	0.65	−0.6
20	Less time for self-care due to digital work	3.17	1.39	0.66	0.49	0.5	−0.55

Digital Fatigue (Items 1–5)

This subscale assessed cognitive and physical exhaustion from digital exposure. Participants reported experiencing mental exhaustion associated with electronic health records (EHRs). The item “I feel mentally exhausted after prolonged use of electronic health records” received a mean score of 3.41 (SD = 1.27), showing a strong correlation with the overall Digital System Challenges (DSC) scale (r = 0.71, *p* < 0.001), as well as significant associations with burnout (r = 0.67, *p* < 0.001) and perceived stress (r = 0.59, *p* < 0.001). Similarly, the item “Using multiple digital platforms in my work is overwhelming” had a mean of 3.35 (SD = 1.33), with strong associations to the DSC (r = 0.69, *p* < 0.001) and perceived stress (r = 0.55, *p* < 0.001).

Impact on Clinical Focus and Job Satisfaction

The item “Digital tasks reduce my focus on direct patient care” was rated at M = 3.38 (SD = 1.22), strongly correlated with the DSC score (r = 0.74, *p* < 0.001) and inversely correlated with job satisfaction (r = −0.58, *p* < 0.001).

Physical Strain and Documentation Preferences

Physical discomfort related to screen use was reported through the item “I experience physical strain (e.g., headaches, eye fatigue) due to screen exposure” (M = 3.18, SD = 1.36; r = 0.63, *p* < 0.001). Preference for paper-based documentation was captured by the item “I would prefer paper documentation over digital methods” (M = 3.04, SD = 1.41; r = 0.59, *p* < 0.001), with higher agreement among professionals over the age of 45 (t = 2.91, *p* = 0.004).

Technostress

Responses related to technostress revealed high levels of strain. The item “I feel stressed when I have to learn new digital systems” had a mean of 3.56 (SD = 1.30), correlating with DSC (r = 0.73, *p* < 0.001) and burnout (r = 0.65, *p* < 0.001). The item “Frequent software updates disrupt my workflow” had a mean of 3.42 (SD = 1.29; r = 0.70, *p* < 0.001), and “Technical problems with digital systems cause frustration” was rated at M = 3.49 (SD = 1.34; r = 0.76, *p* < 0.001). The frequency of digital alerts was addressed in the item “I receive too many digital alerts or notifications during work” (M = 3.29, SD = 1.37; r = 0.68, *p* < 0.001), correlating with perceived stress (r = 0.61, *p* < 0.001). Inadequate training was also noted (M = 3.11, SD = 1.42; r = 0.66, *p* < 0.001), with the strongest association observed with perceived role overload (r = 0.62, *p* < 0.001).

Digital Disengagement

Participants indicated emotional detachment associated with digital systems. The item “Digital tools reduce the human connection in my interactions with patients” had a mean score of 3.52 (SD = 1.27), correlating with DSC (r = 0.71, *p* < 0.001) and negatively with job satisfaction (r = −0.64, *p* < 0.001). Related items also reflected similar experiences: “I spend more time on screens than interacting with patients” (M = 3.33, SD = 1.34; r = 0.70, *p* < 0.001), “I sometimes feel emotionally detached due to digital documentation” (M = 3.14, SD = 1.38; r = 0.65, *p* < 0.001), “Telepsychiatry feels less personal than in-person care” (M = 3.27, SD = 1.31; r = 0.67, *p* < 0.001), and “Technology limits my ability to provide holistic care” (M = 3.10, SD = 1.35; r = 0.61, *p* < 0.001).

Work–Life Digital Boundaries

Digital systems were reported to intrude on personal time. The item “I check work-related emails or messages outside of my shifts” received the highest average score (M = 3.61, SD = 1.36), strongly correlated with DSC (r = 0.75, *p* < 0.001) and negatively with work–life balance satisfaction (r = −0.68, *p* < 0.001). Respondents also agreed with “Digital communications make it hard to disconnect from work” (M = 3.47, SD = 1.33; r = 0.72, *p* < 0.001) and “Digital demands negatively affect my sleep” (M = 3.28, SD = 1.37; r = 0.68, *p* < 0.001), the latter correlating with insomnia symptoms (r = 0.58, *p* < 0.001). Additional items included “I feel expected to be digitally available even outside work hours” (M = 3.50, SD = 1.34; r = 0.73, *p* < 0.001) and “Digital work reduces the time I have for self-care” (M = 3.17, SD = 1.39; r = 0.66, *p* < 0.001).

## 4. Discussion

This study aimed to develop and validate the Digital Stress Scale (DSC) and to assess digital stress levels among mental health professionals across four dimensions: digital fatigue, technostress, digital disengagement, and work–life digital boundaries. The findings support the reliability, structural validity, and practical relevance of the DSC in capturing the nuanced impact of digitalization on psychiatric nursing practice. Each domain of the scale revealed critical stressors that resonate with the emerging literature on digital health and workforce well-being. While digital systems aim to enhance documentation efficiency, remote access, and communication, their unintended psychological consequences—particularly in emotionally intensive roles such as mental healthcare—warrant close examination. A balanced view is needed to preserve the benefits while mitigating the burden.

### 4.1. Digital Fatigue and Cognitive Overload

The DSC identified digital fatigue as the most potent predictor of overall digital stress. Items such as “I feel mentally exhausted after prolonged EHR use” showed high ratings and strong correlations with burnout and job dissatisfaction. These findings mirror the results of Ayyagari et al. [1] and Demerouti et al. [2], who noted that digital fatigue in healthcare is often the result of continuous cognitive demands, screen exposure, and multitasking with electronic systems. Cognitive overload, particularly in high-stakes environments such as psychiatric care, can impair decision-making and reduce the capacity for empathetic interaction [3]. Health professionals frequently reported that digital documentation reduced their attentional focus on therapeutic engagement—a finding consistent with studies showing that screen-based clinical workflows can disrupt emotional presence in mental health settings [4].

### 4.2. Technostress and System Complexity

The second dimension, technostress, also emerged as a major contributor to overall digital stress. Items such as “I feel stressed when I have to learn new digital systems” and “Frequent software updates disrupt my workflow” reflected the health professionals’ difficulties in keeping pace with evolving technology. This aligns with the foundational work of Tarafdar et al. [5], who identified technostress creators including techno-overload, techno-insecurity, and techno-complexity. More recent studies in the post-COVID-19 era highlight that while digital innovations in healthcare offer significant utility, the speed of adoption often surpasses the pace of adequate training, leading to stress, frustration, and task ineffectiveness [6,7]. In our study, a lack of digital preparedness was correlated with increased feelings of burnout and perceived role overload, reinforcing the urgent need for structured digital literacy programs tailored to mental health professionals.

### 4.3. Digital Disengagement and Therapeutic Erosion

A particularly novel contribution of the DSC is its inclusion of digital disengagement, a dimension capturing the emotional and relational disconnect that can occur when patient interactions are mediated by digital tools. Items such as “Telepsychiatry feels less personal than in-person care” and “I feel emotionally detached due to digital documentation” revealed moderate-to-high levels of disengagement, especially among younger health professionals. These results are in line with recent qualitative and mixed-methods studies suggesting that while digital tools facilitate access to care, they can compromise the richness of therapeutic interactions [8,9]. For instance, Moloney et al. [10] documented how telepsychiatry limits the ability of clinicians to assess non-verbal cues and build emotional rapport. Our findings extend this work by quantitatively linking digital disengagement to decreased job satisfaction and compassion fatigue, further emphasizing the need to balance technological efficiency with human-centered care design.

### 4.4. Work–Life Digital Boundaries and Hyperconnectivity

The fourth domain, work–life digital boundaries, revealed a high prevalence of digital spillover into personal time. The highest scoring item—“I check work-related emails or messages outside of my shifts”—was reported by over 70% of health professionals as a regular experience. This is consistent with studies by Derks and Bakker [11], who found that after-hours connectivity contributes significantly to emotional exhaustion and work–life conflict. Our findings align with the emerging discourse on “technological tethering” in healthcare, wherein the digital workplace extends beyond physical settings, often without institutional boundaries [12]. Given that work–life balance is a critical determinant of mental health and retention [13], these findings suggest a need for policy interventions, such as “digital disconnection” protocols and after-hours communication limits.

### 4.5. Overall Scale Performance and Implications

The DSC demonstrated excellent internal consistency (α = 0.87), strong factor structure through EFA and CFA, and robust convergent validity with external indicators of stress, burnout, and job satisfaction. These psychometric properties suggest that the scale is both theoretically sound and practically useful for workforce assessments. The predictive value of digital fatigue and technostress in the regression model supports prior frameworks, such as the Job Demands–Resources Model, which posits that excessive digital demands can deplete psychological resources and reduce work engagement [14]. Our findings also reinforce the need to include emotional and relational components—captured here through digital disengagement and work–life boundaries—in future models of occupational stress in healthcare. While Pearson’s r was used to assess convergent validity in this initial validation, we acknowledge that some of the observed correlations fall just below the commonly recommended threshold of 0.70.

Given the ordinal nature of the DSC items, future analyses will employ Spearman’s rho to obtain more appropriate estimates of convergent validity.

## 5. Conclusions

The DSC appears to be a valid and reliable tool for assessing digital stress among mental health professionals within the Greek and Cypriot contexts. Further research is needed to establish its cross-cultural applicability. By identifying key stressors such as digital fatigue, technostress, digital disengagement, and work–life digital boundaries, this tool provides valuable insights into the challenges faced by health professionals in the digital era. Addressing these stressors through targeted interventions and supportive policies is essential to enhance nurse well-being and maintain the quality of psychiatric care. Future research should focus on longitudinal studies to further explore the impact of digital stress and evaluate the effectiveness of mitigation strategies.

### Strengths and Limitations

The primary strength of this study lies in the development of a psychometrically robust and context-specific tool, grounded in both theory and qualitative insights from clinical practice. The sample size was adequate for factor analysis, and the diversity of settings (inpatient and outpatient) enhances generalizability. However, several limitations warrant consideration. First, the cross-sectional design limits causal inferences regarding the relationship between digital stress and psychological outcomes. Second, although participants represented multiple regions, cultural variations in digital engagement may influence perceptions of stress. Third, while the regression model explained a high proportion of variance, further studies should explore longitudinal patterns and the role of moderating variables such as organizational support or digital literacy. Another limitation of this study relates to the cultural and institutional context in which the data were collected. All participants were mental health professionals based in Greece and Cyprus—two countries that share common cultural values, language, and comparable healthcare systems. While this consistency offers contextual clarity, it also limits the generalizability of the findings. The applicability of the DSC may be influenced by differences in digital infrastructure, institutional support, and cultural attitudes toward work–life balance and emotional expression. To ensure broader relevance, future research should focus on cross-cultural validation of the scale, examining its structural consistency and contextual fit in diverse international settings. It is important to emphasize that the cross-sectional nature of this study precludes any conclusions about causality. While significant associations were identified between digital stress and mental health outcomes—such as burnout and job dissatisfaction—these relationships should be interpreted as correlational. Further longitudinal and experimental research is necessary to explore the directionality and potential causal pathways underlying these findings.

## Figures and Tables

**Table 1:** Participant demographics.

Variable	Value
Total Participants	423
Mean Age (years)	39.4
Standard Deviation (Age)	6.2
Gender—Female (%)	71.4%
Gender—Male (%)	28.6%
Mean Years of Experience	9.8
Standard Deviation (Experience)	5.1
Inpatient Setting (%)	62%
Outpatient Setting (%)	38%

**Table 2:** Variance explained by each factor (EFA).

Factor	Eigenvalue	% of Variance	Cumulative %
Digital Fatigue	5.8	29.0%	29.0%
Technostress	4.0	20.0%	49.0%
Digital Disengagement	2.7	13.4%	62.4%
Work–Life Digital Boundaries	2.0	10.0%	72.4%

**Table 3:** Fit indices for Confirmatory Factor Analysis.

Fit Index	Value	Acceptable Threshold
CFI	0.965	>0.90
TLI	0.943	>0.90
RMSEA	0.038	<0.08
SRMR	0.041	<0.08

**Table 4:** EFA and CFA factor loadings for DSC items.

Item No.	Subscale	EFA Loading	CFA Loading
1	Digital Fatigue	0.78	0.74
2	Digital Fatigue	0.75	0.72
3	Digital Fatigue	0.81	0.78
4	Digital Fatigue	0.72	0.69
5	Digital Fatigue	0.69	0.67
6	Technostress	0.82	0.79
7	Technostress	0.79	0.76
8	Technostress	0.84	0.82
9	Technostress	0.76	0.74
10	Technostress	0.74	0.71
11	Digital Disengagement	0.8	0.77
12	Digital Disengagement	0.77	0.75
13	Digital Disengagement	0.73	0.71
14	Digital Disengagement	0.78	0.76
15	Digital Disengagement	0.71	0.69
16	Work–Life Boundaries	0.83	0.81
17	Work–Life Boundaries	0.79	0.78
18	Work–Life Boundaries	0.76	0.74
19	Work–Life Boundaries	0.81	0.8
20	Work–Life Boundaries	0.74	0.72

**Table 5:** Multiple linear regression.

Predictor	Beta Coefficient (β)	Standard Error	t-Value	*p*-Value
Digital Fatigue	0.35	0.04	8.75	<0.001
Technostress	0.28	0.05	5.6	<0.001
Digital Disengagement	0.22	0.04	5.5	<0.001
Work–Life Digital Boundaries	0.26	0.05	5.2	<0.001

## Data Availability

Data available upon request.

## Appendix A. Full Version of the Digital Stress Scale (DSC)

The following 20 items constitute the final version of the Digital Stress Scale (DSC). Each item is rated on a 5-point Likert scale ranging from 1 = “Strongly disagree” to 5 = “Strongly agree”.

### Appendix A.1. Digital Fatigue

1. I feel mentally exhausted after prolonged use of electronic health records (EHRs).
2. Using multiple digital platforms in my work is overwhelming.
3. Digital tasks reduce my focus on direct patient care.
4. I experience physical strain (e.g., headaches, eye fatigue) due to screen exposure.
5. I would prefer paper documentation over digital methods.

### Appendix A.2. Technostress

6. I feel stressed when I have to learn new digital systems.
7. Frequent software updates disrupt my workflow.
8. Technical problems with digital systems cause frustration.
9. I receive too many digital alerts or notifications during work.
10. I have not received adequate training in using digital tools.

### Appendix A.3. Digital Disengagement

11. Digital tools reduce the human connection in my interactions with patients.
12. I spend more time on screens than interacting with patients.
13. I sometimes feel emotionally detached due to digital documentation.
14. Telepsychiatry feels less personal than in-person care.
15. Technology limits my ability to provide holistic care.

### Appendix A.4. Work–Life Digital Boundaries

16. I check work-related emails or messages outside of my shifts.
17. Digital communications make it hard to disconnect from work.
18. Digital work demands negatively affect my sleep.
19. I feel expected to be digitally available even outside work hours.
20. Digital work reduces the time I have for self-care.
